# Supplementary material for: Nonmagnetic framboid and associated iron nanoparticles with a space-weathered feature from asteroid Ryugu
Source: Nat Commun. 2024 Apr 29;15:3493. doi: 10.1038/s41467-024-47798-0 (PMC11059182; doi:10.1038/s41467-024-47798-0)
Supplement: Supplementary file 1 — Supplementary Information [file 41467_2024_47798_MOESM1_ESM.pdf]

Supplementary Information for

Nonmagnetic framboid and associated iron nanoparticles with a space-weathered feature from asteroid Ryugu

Yuki Kimura, Takeharu Kato, Satoshi Anada, Ryuji Yoshida, Kazuo Yamamoto, Toshiaki Tanigaki, Tetsuya Akashi, Hiroto Kasai, Kosuke Kurosawa, Tomoki Nakamura, Takaaki Noguchi, Masahiko Sato, Toru Matsumoto, Tomoyo Morita, Mizuha Kikuiri, Kana Amano, Eiichi Kagawa, Toru Yada, Masahiro Nishimura, Aiko Nakato, Akiko Miyazaki, Kasumi Yogata, Masanao Abe, Tatsuaki Okada, Tomohiro Usui, Makoto Yoshikawa, Takanao Saiki, Satoshi Tanaka, Fuyuto Terui, Satoru Nakazawa, Hisayoshi Yurimoto, Ryuji Okazaki, Hikaru Yabuta, Hiroshi Naraoka, Kanako Sakamoto, Sei-ichiro Watanabe, Yuichi Tsuda, and Shogo Tachibana

Table S1. Iron/nickel atomic ratio (at%) of particles 1–11 in Fig. S4.

	1	2	3	4	5	6	7	8	9	10	11
Fe	100	100	100	100	100	100	100	95.8	96.0	92.0	87.5
Ni	0	0	0	0	0	0	0	4.2	4.0	8.0	12.5

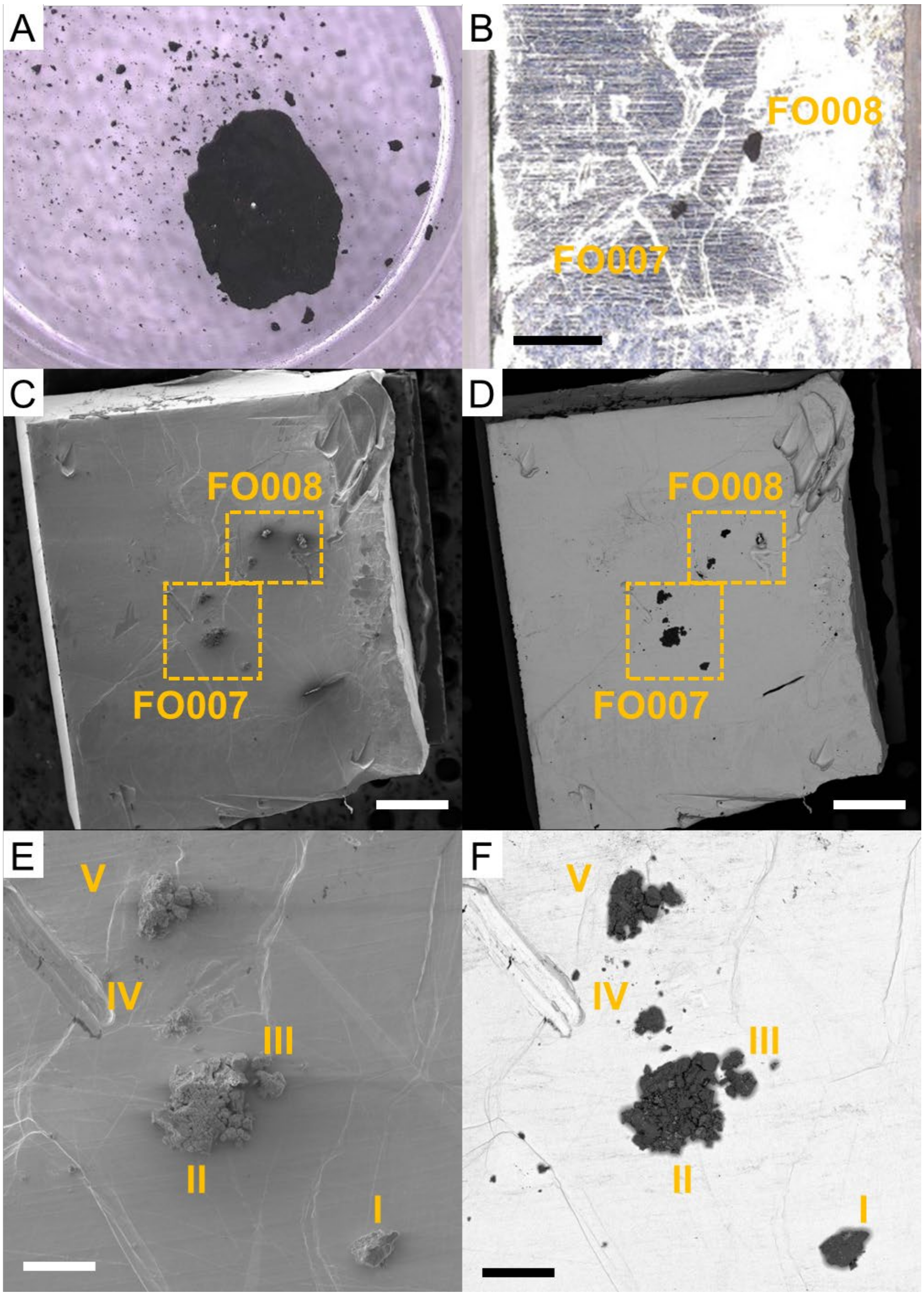

Fig. S1. Samples A0064–FO007 and A0064–FO008 of asteroid Ryugu. A. Optical micrograph of the A0064 particle (~3 mm size) in a glass storage vessel. **B**. Fragments FO007 and FO008 on an indium plate. **C.** Secondary electron image obtained using a tabletop scanning electron microscope (JCM-7000, NeoScope, JEOL Ltd., Tokyo). **D**. Corresponding backscatter electron image. Because both pieces were composed of several particles, particles within the corresponding rectangles were attributed to FO007 or FO008, respectively. **E**. Enlarged secondary electron image of the piece FO007. **F**. Corresponding backscatter electron image of E. The scale bars are 500 μm for B–D and 100 μm for E and F.

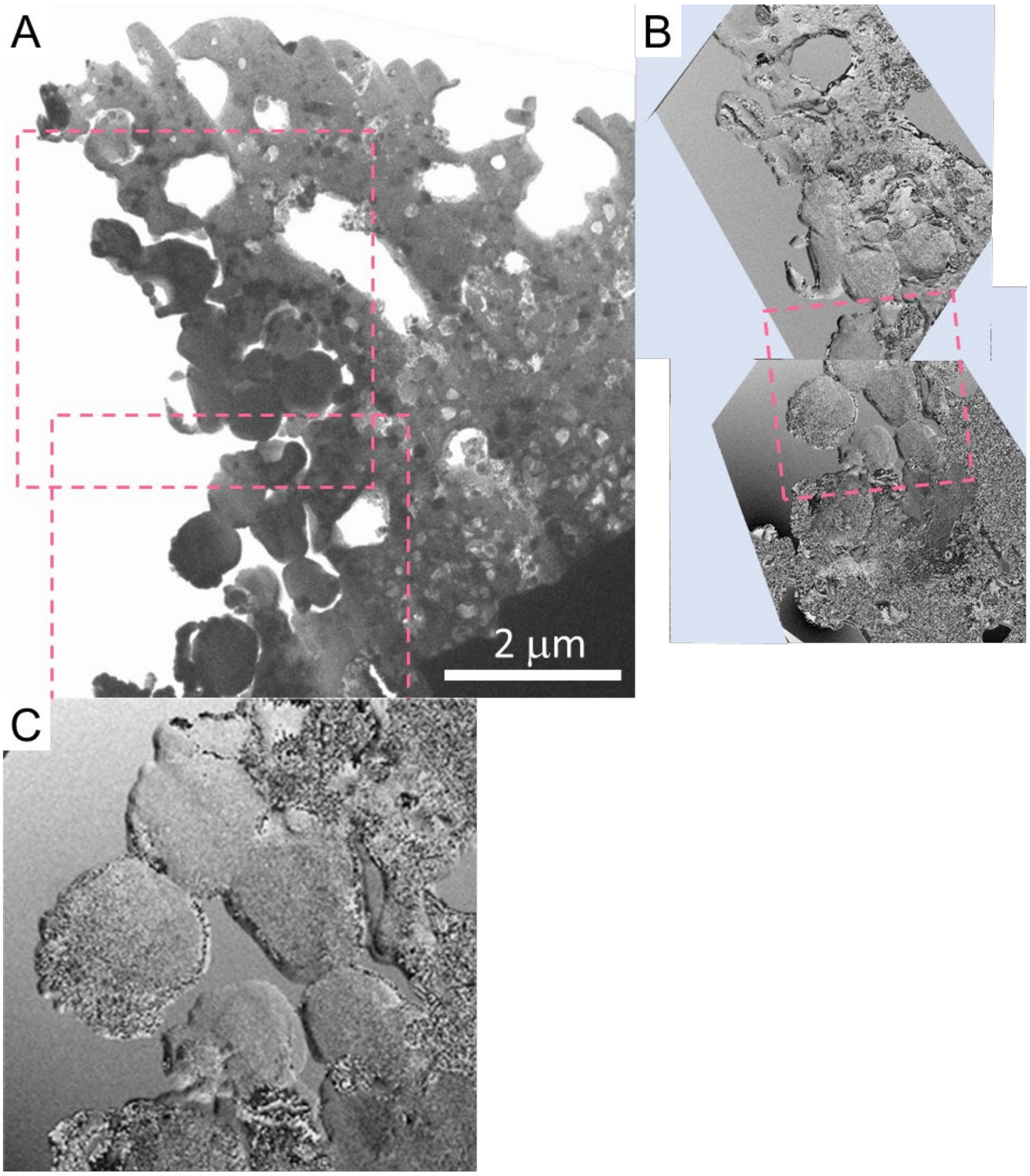

Fig. S2. Pseudo-framboid in FIBii. A. Bright-field TEM image of a thin section extracted from Position ii (FIBii) on the surface of particle A0064–FO007–I in Fig. 1A. **B**. Combined magnetic-flux-distribution (two-times phase-amplified phase reconstruction) image of the rectangular regions in A. **C**. Magnified image of the rectangular region in B.

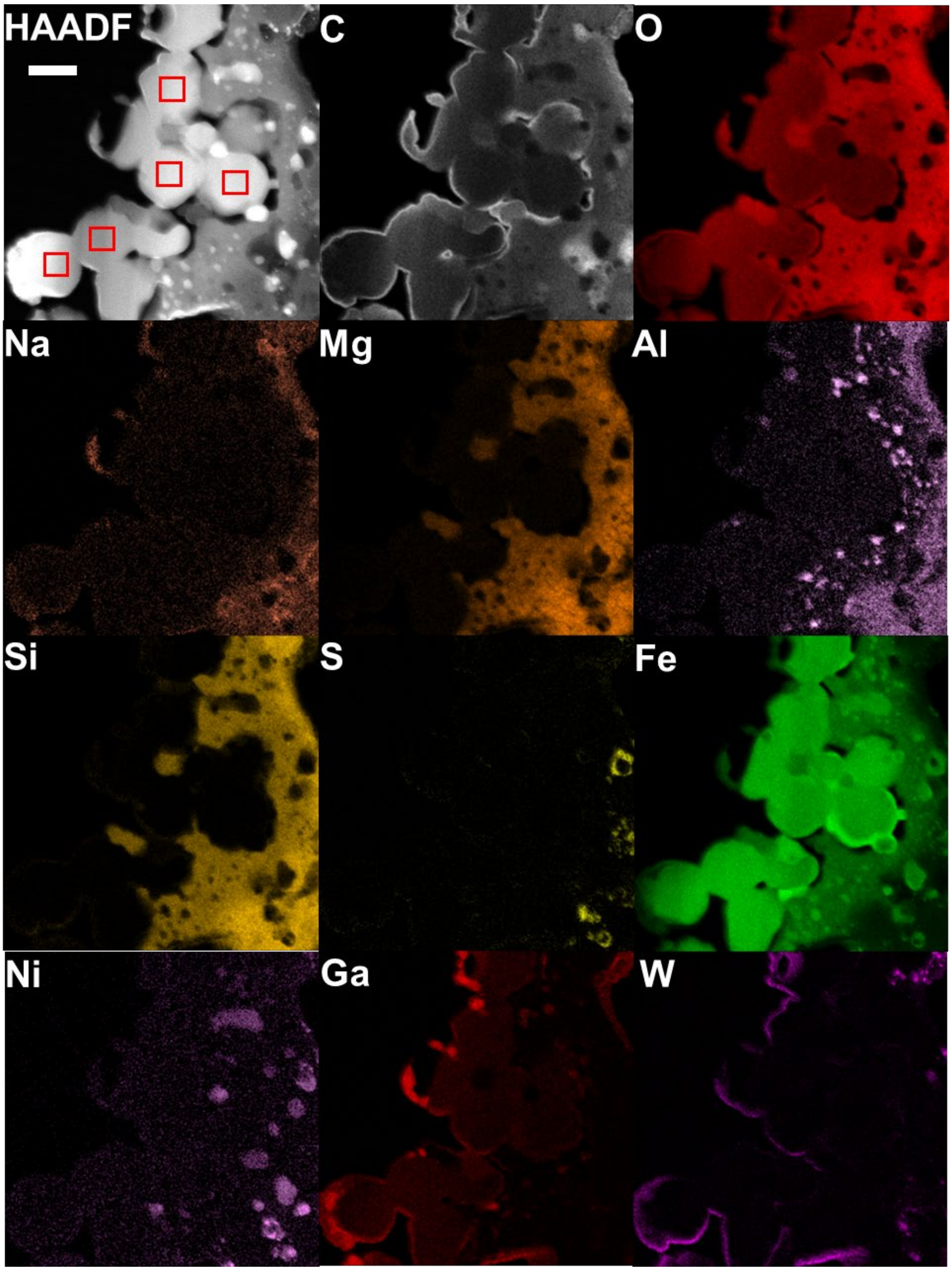

Fig. S3. Magnified HAADF–STEM image of the pseudo-magnetite in FIBii shown in Fig. 1, and corresponding elemental mapping. The five squares in the HAADF image indicate positions at which the averaged elemental ratio of iron and oxygen of the pseudo-magnetite were obtained. The original surface is at the left-hand side of the image. Note that a 30-nm-thick layer of carbon was deposited onto each face of the thin section. Oxygen in the surface-rounded particles and sodium, magnesium, silicon, and sulfur in the matrix just beneath the rounded particle show depletion. Aluminum is round in shape, appearing to have been melted and solidified at some stage, and is located away from the surface. The widespread distribution of iron in the matrix surrounding the pseudo-magnetite exclusively is likely to have been the result of release from the framboid. The scale bar is 500 nm.

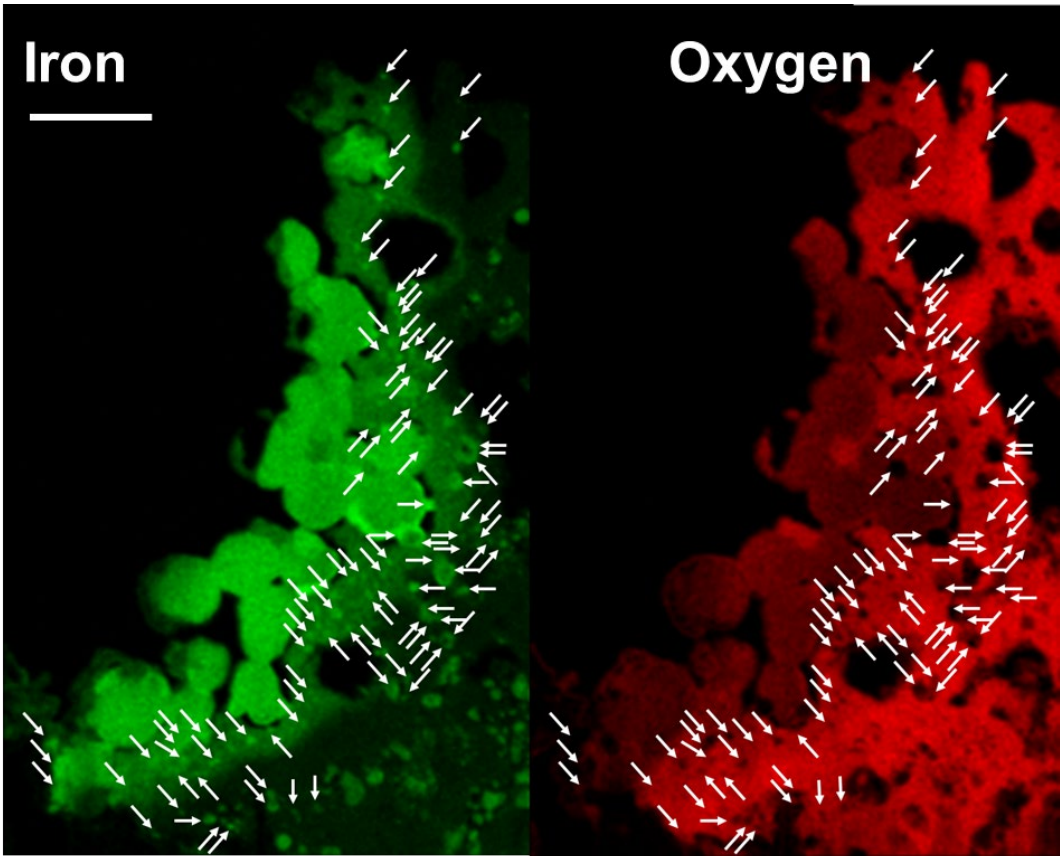

Fig. S4. Metallic iron particles in the iron leaching area. Arrows indicate oxygen-absent iron-rich regions, which are metallic iron particles around the pseudo-magnetite in FIBii shown in Fig. 2C. The scale bar is 500 nm.

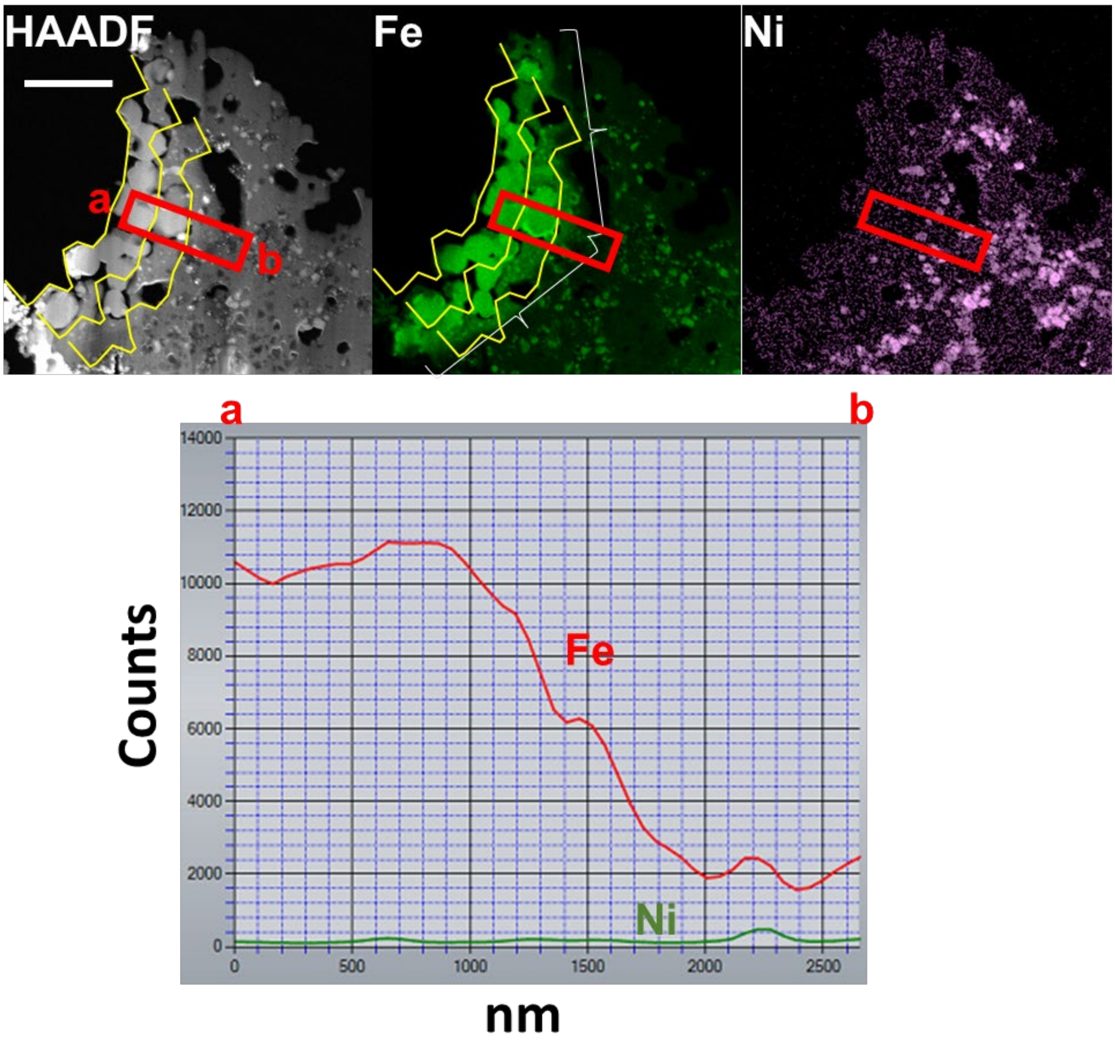

Fig. S5. Line profiles of iron and nickel from the surface to the interior. The profiles correspond to the red box region from a to b in the HAADF–STEM and elemental mapping images. The scale bar is 2 μm.

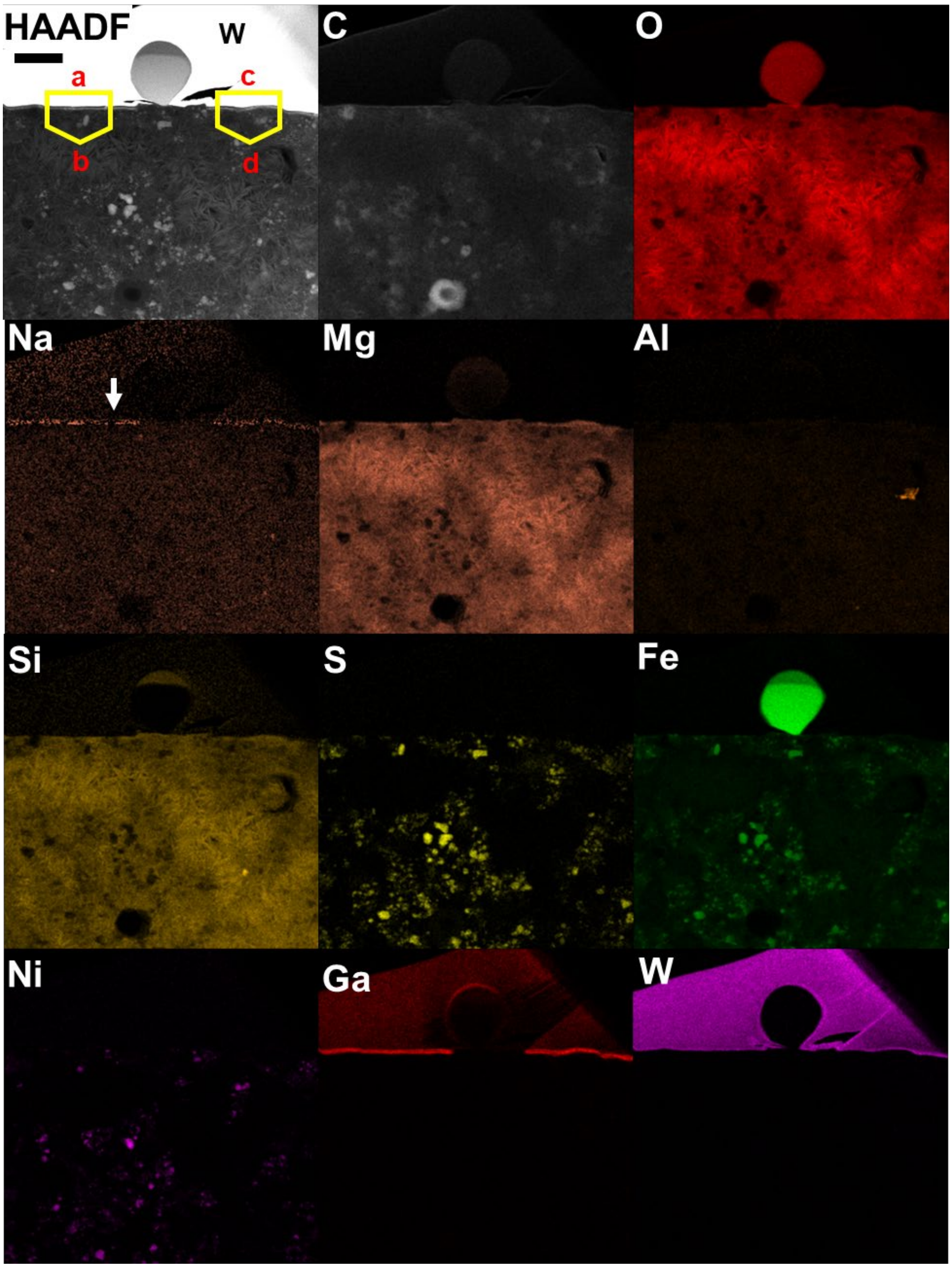

Fig. S6. HAADF–STEM image of the pseudo-magnetite in FIBiii shown in Fig. 6, and the corresponding elemental mapping. This section corresponds to Region iii in Fig. 1A. Note that a 30-nm-thick layer of carbon was deposited onto each face of the thin section. No depletion of sodium, magnesium, silicon, or sulfur in the matrix at the surface was detectable. The intense signal of sodium indicated by the white arrow is an artefact caused by the Ga-L line. The scale bar is 1 μm.

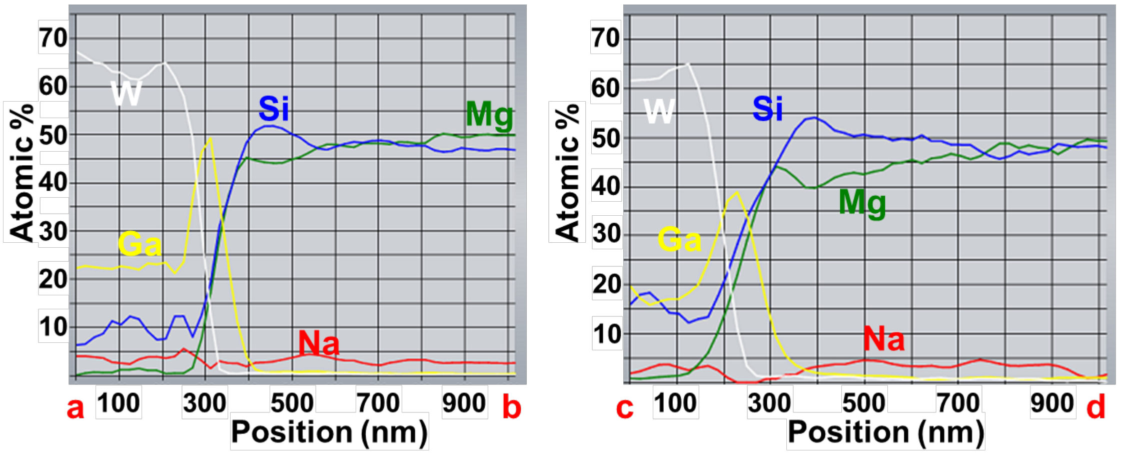

Fig. S7. Line profiles of silicon, sodium, magnesium, gallium, and tungsten from the surface to the interior. The left- and right-hand panels show line profiles in the regions from a to b and c to d, respectively, in Fig. S6. The Si-K line overlaps the W-M line, and the Na-K line overlaps the Ga-L line, resulting in a profile with a high silicon concentration inside the tungsten deposition and a high sodium concentration on the surface because of poor peak separation with gallium. The particle surfaces are at ~350 nm and ~300 nm for the left- and right-hand panels, respectively.

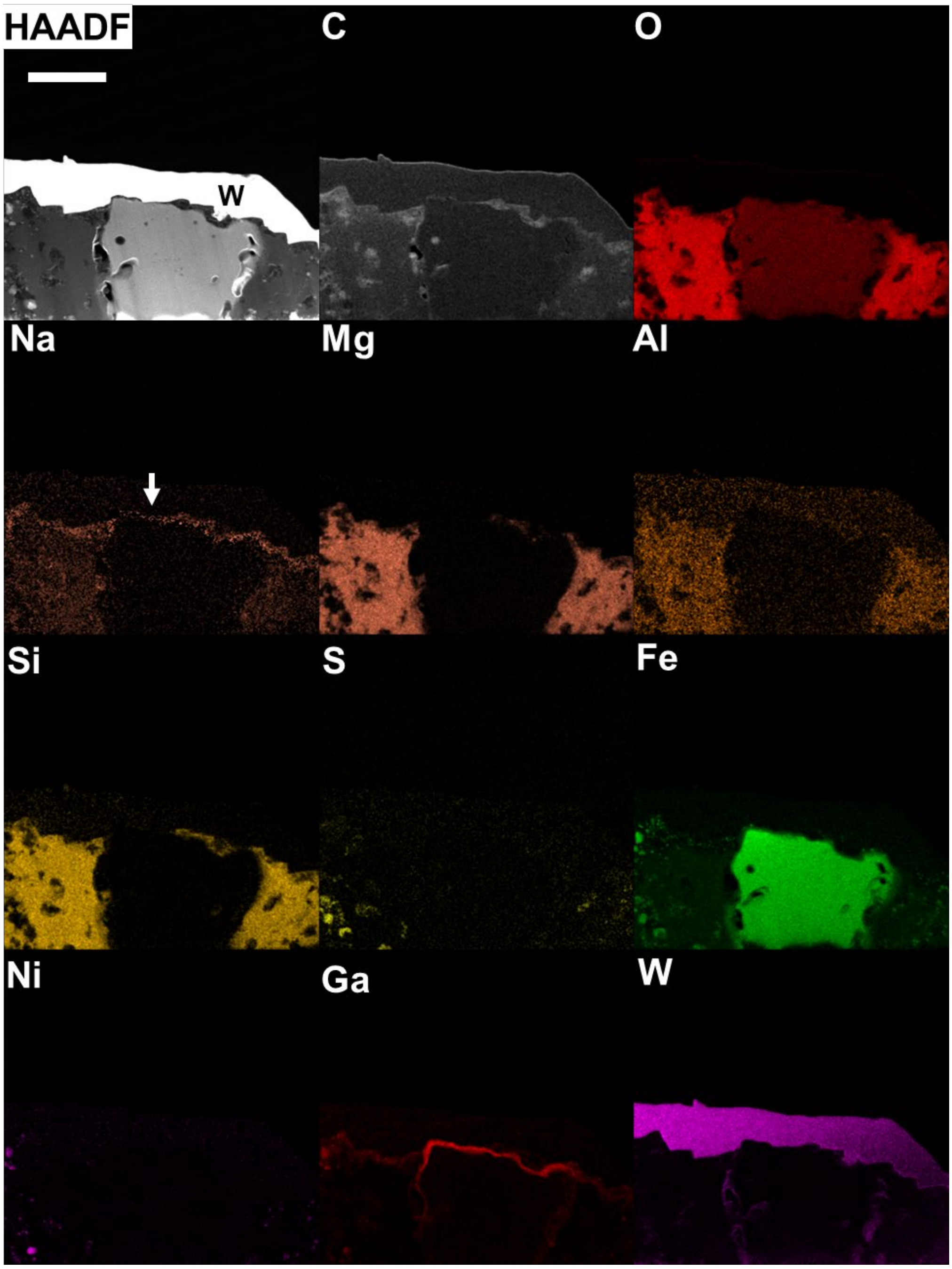

Fig. S8. HAADF–STEM image of the pseudo-magnetite in FIBiv shown in Fig. 6 and the corresponding elemental mapping. This section corresponds to region iv in Fig. 1A. Note that a 30-nm-thick layer of carbon was deposited onto each face of the thin section. No depletion of sodium, magnesium, silicon, or sulfur in the matrix at the surface was detectable. The intense signal for sodium, indicated by the white arrow, is an artefact caused by the Ga-L line. The scale bar is 2 μm.

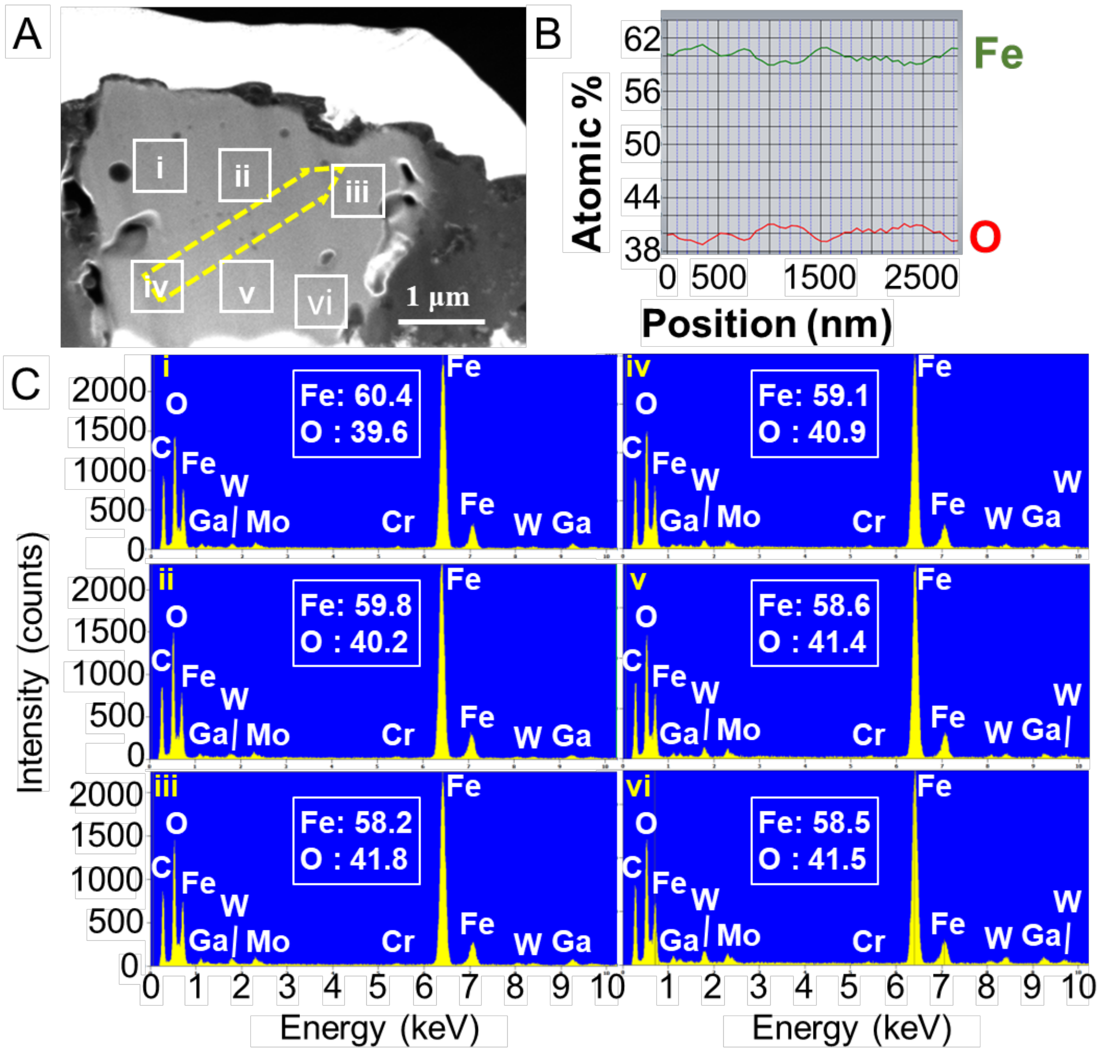

Fig. S9. Iron-oxygen atomic ratio of the pseudo-magnetite in FIBiv shown in Figs. 3 and S8. **A**. HAADF-STEM image. This section corresponds to region iv in Fig. 1A. **B**. Line profiles of Fe and O from the bottom left to the upper right as shown by dashed arrow in A. **C**. EDS data corresponds to the boxes i-vi in A. Numbers in the white boxes show normalized atomic % of Fe and O. Signal peaks of C, Ga, W, Mo and Cr are originating from deposited carbon layer to prevent electrical charging, Ga− ion beam for FIB machining, deposited film onto the processed area to protect original surface, TEM grid, and pole piece of TEM, respectively.

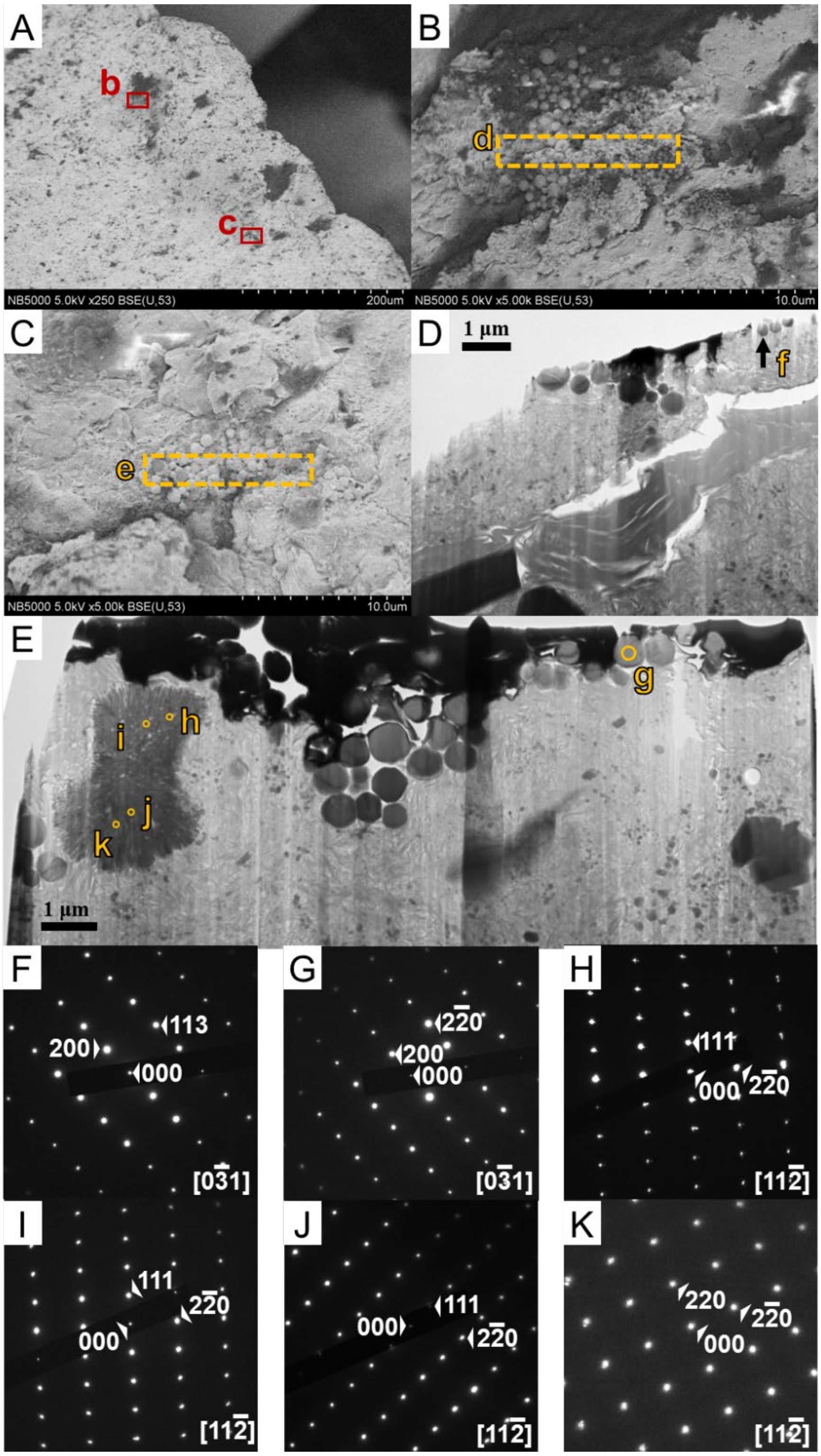

Fig. S10. Magnetite on the surface of the space-weathered particle A0067. A. SEM backscattered electron image. **B** and **C**. Magnified image of the rectangular regions b and c in A. **D** and **E**. Bright-field TEM images of thin sections prepared from boxes d in B and e in C, respectively. The corresponding elemental mappings are shown in SI, Figs. S12 and S13. **F–K**. SAED patterns corresponding to arrow f in D and circles g–k in E, respectively. White triangles with *hkl* indices indicate the corresponding diffraction spots.

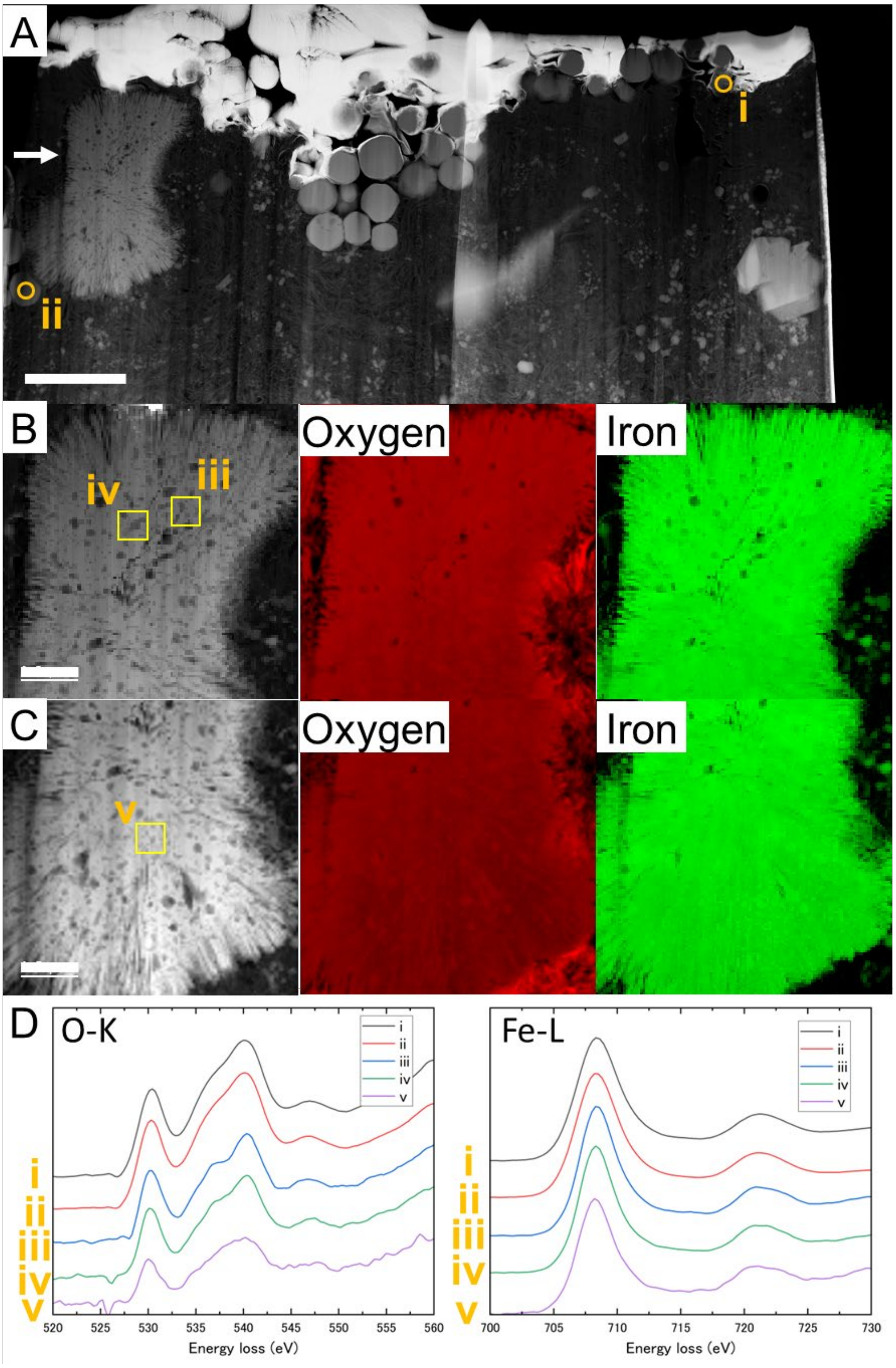

Fig. S11. Magnetite on the surface of the space-weathered particle A0067. A. HAADF–STEM image of a thin section corresponding to E in SI, Fig. S10: two images have been combined. **B** and **C**. Magnified image of the upper and lower parts of the magnetite, respectively, indicated by the arrow in A, and the corresponding EELS maps of oxygen (red) and iron (green). **D**. EELS data of oxygen and iron obtained from the circles b and d in A. Scale bars are 2 µm for A and 500 nm for B and C.

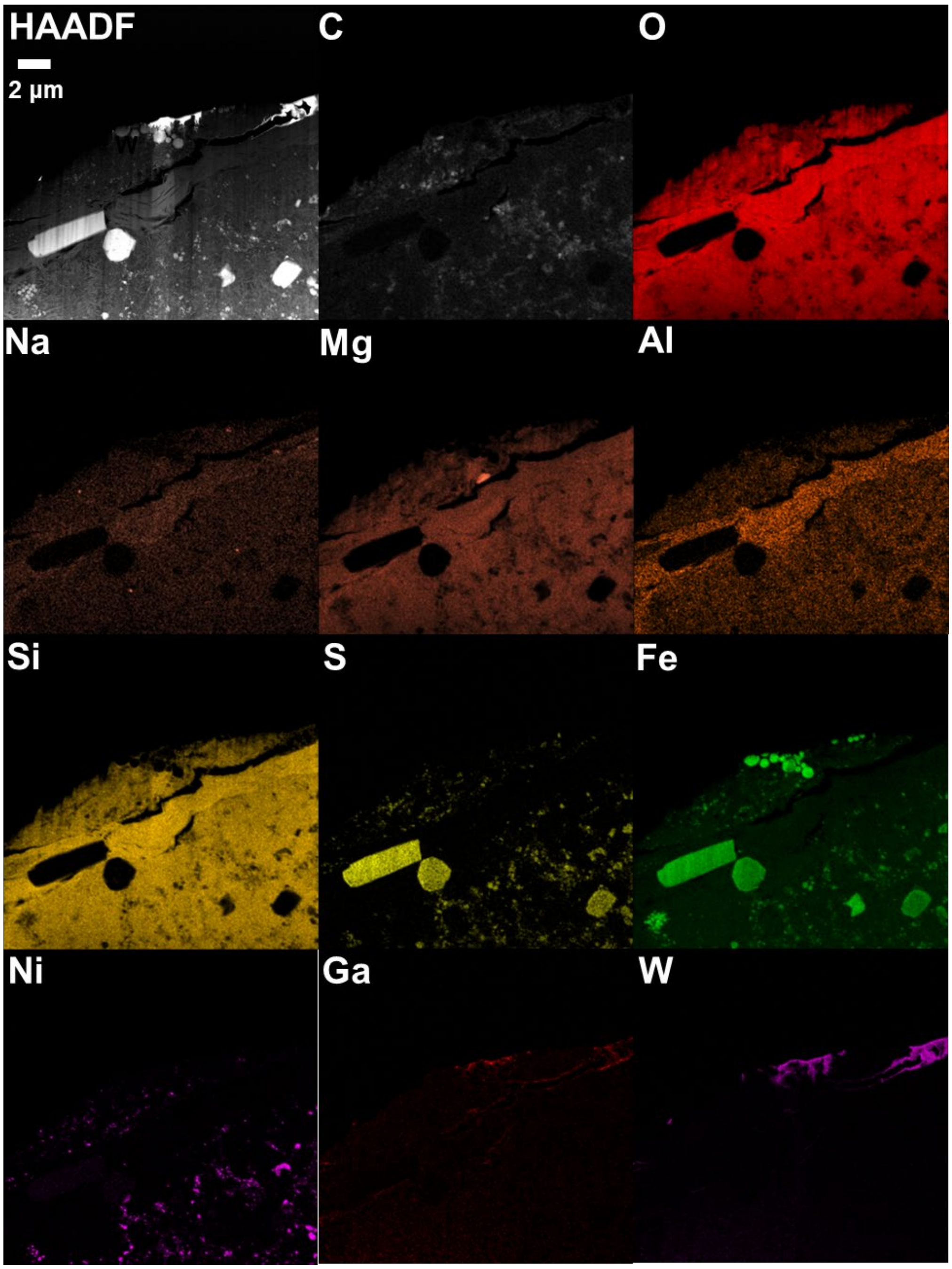

Fig. S12. HAADF-STEM image and corresponding elemental mapping of a thin section of the space-weathered particle A0067. This thin section corresponds to Fig. S10D. The scale bar is 2 µm.

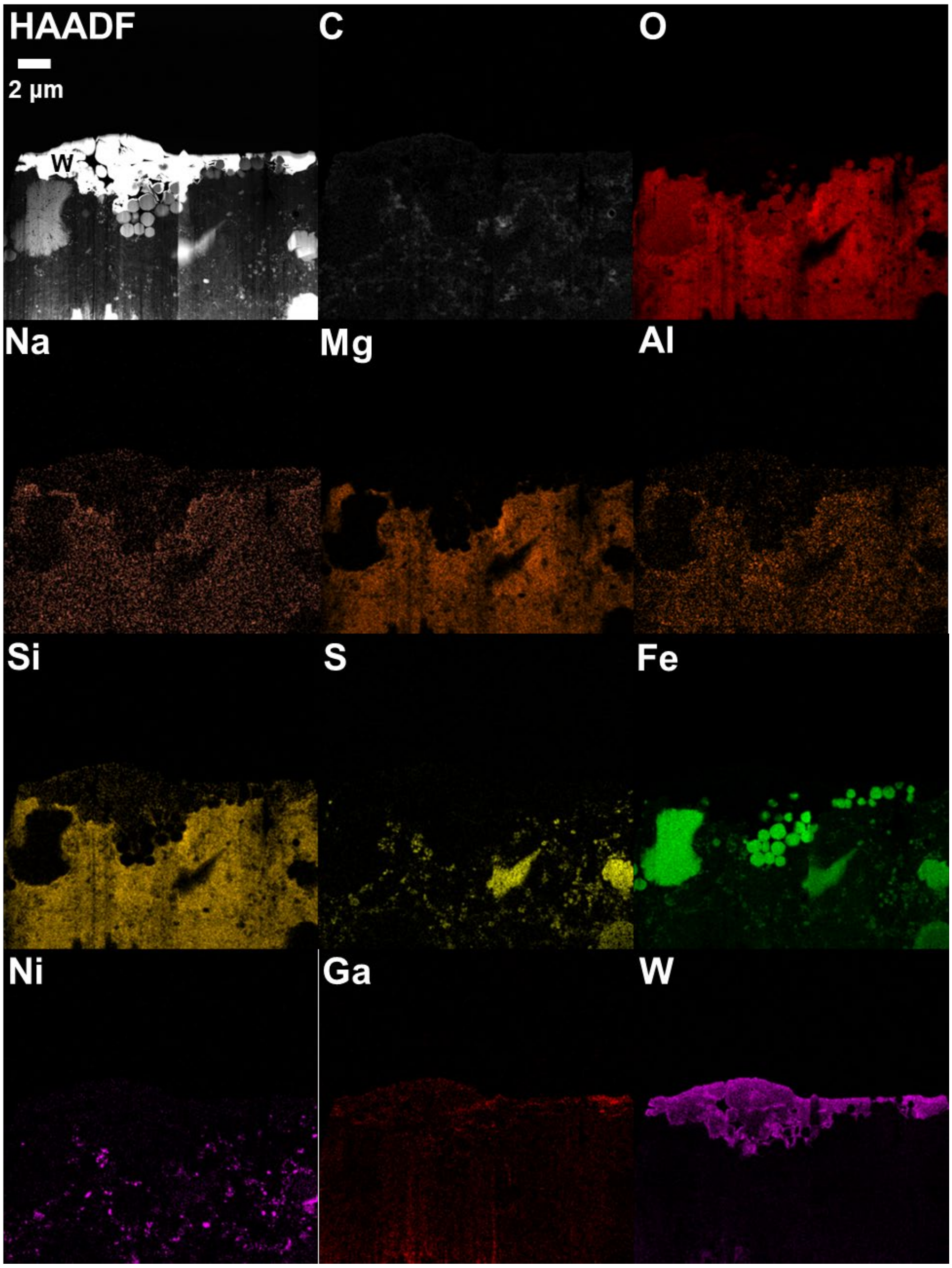

Fig. S13. HAADF–STEM image and corresponding elemental mapping of a thin section of the space-weathered particle A0067. This thin section corresponds to Fig. S10E. The scale bar is 2 µm.

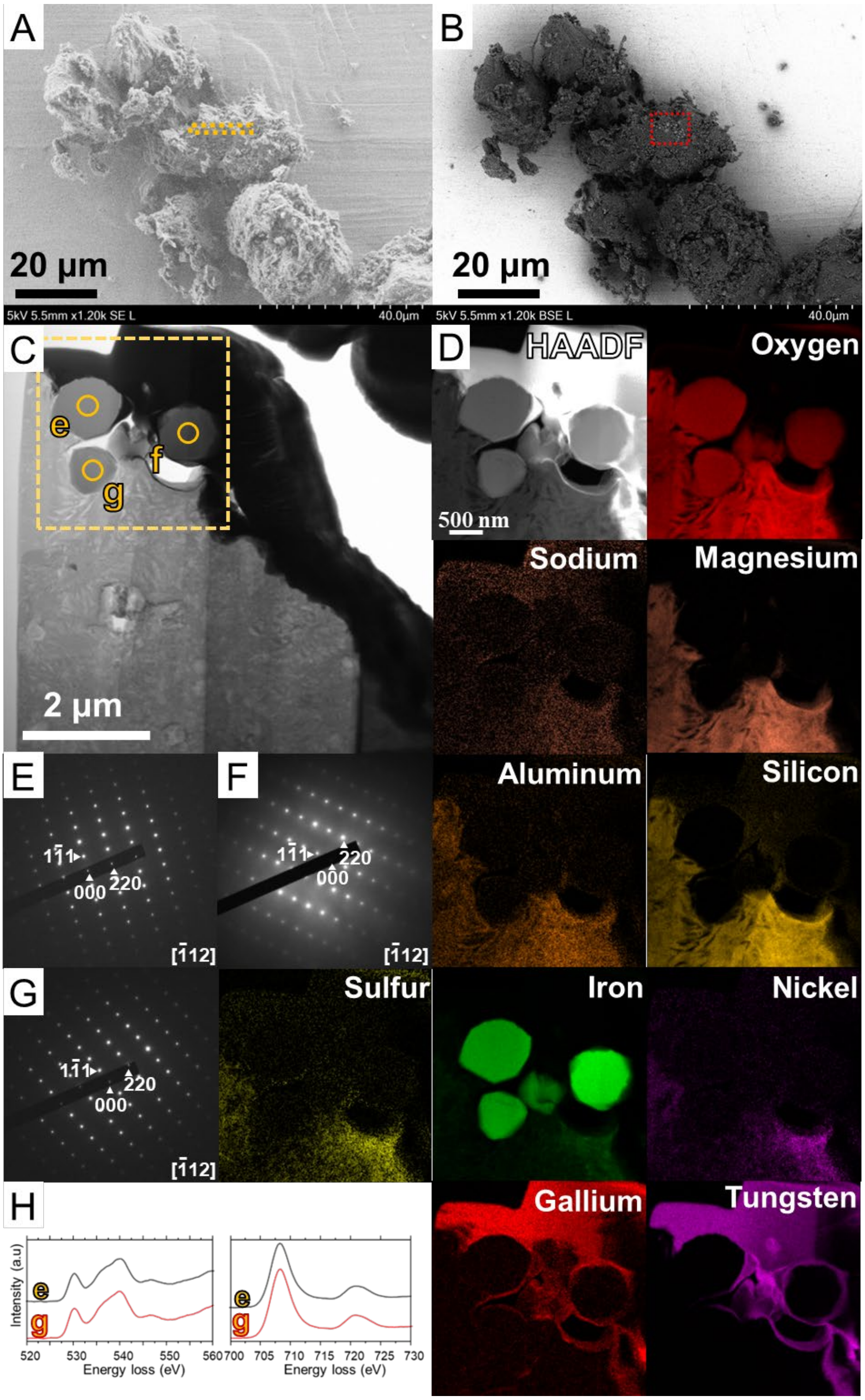

Fig. S14. Framboid in the Orgueil meteorite heated to 773 K for 500 h. A. Secondary electron image. **B**. Corresponding backscatter electron image. **C.** Bright-field TEM image of an ultrathin section prepared from the box in A. **D**. HAADF–STEM image and elemental map corresponding to the box in C. **E-G**. SAED patterns obtained from e–g in C. **H**. EELS data of oxygen and iron obtained from regions e and g in C.

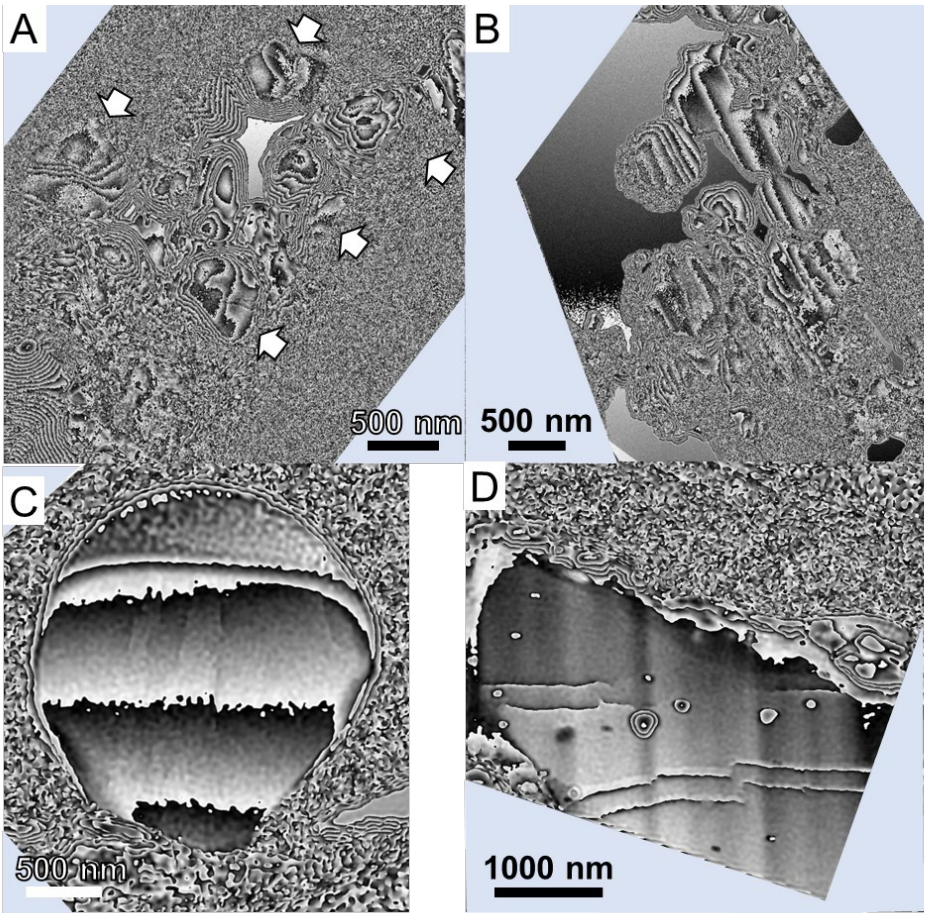

Fig. S15. Internal potential images of framboids. All images are two times the phase-amplified reconstruction. **A.** Framboidal magnetites correspond to those in Figs. 2A and 2B. Because the particles are spherical, even though the center is flattened by FIB processing, there is thickness inhomogeneity due to roundness in the surrounding area. As a result, some particles have smaller integrated internal potentials near the edges. These potentials affect the magnetic field strength quantitatively; however, they do not generate the vortex magnetic structure seen in Fig. 2B artificially. For example, although the particles indicated by the arrows have a clear vortex structure in Fig. 2B, the internal potentials show homogeneous contrasts. **B**. Pseudo-magnetite particle corresponding to that in Fig. 2D. The periodic change in contrast (phase difference) is π rad. Assuming that the average internal potential of the particles is 18 V, the phase difference corresponds to a film thickness of ~25 nm. Although the film becomes gradually thinner toward the lower left, this thickness inhomogeneity does not affect the homogeneous contrast result of the magnetic-flux-distribution image because of the absence of a strong magnetic field. **C.** Relatively large pseudo-magnetite with an amorphous iron silicate layer corresponding to that in Figs. 6A and 6E. The change in contrast indicates that the upper portion is ~50 nm thinner than the lower portion. However, because the particles are homogeneously magnetic field-free (Fig. 6E), differences in film thickness do not affect the results. **D**. Pseudo-magnetite corresponding to that in Figs. 6G and 6H. Because phase differences are rarely observed, the thickness of the section is homogeneous within 25 nm.
